# Interpretable prediction of nucleic acid-binding proteins using a protein language model

**DOI:** 10.1093/bioadv/vbag235

**Published:** 2026-08-17

**Authors:** Hanjin Kim, Sung-Gwon Lee, Jooseong Oh, Kee K Kim, Eun-Mi Kim, Chungoo Park

**Affiliations:** School of Biological Science and Technology, Chonnam National University, Gwangju 61186, Republic of Korea; Institute of Systems Biology and Life Science Informatics, Chonnam National University, Gwangju 61186, Republic of Korea; School of Biological Science and Technology, Chonnam National University, Gwangju 61186, Republic of Korea; School of Biological Science and Technology, Chonnam National University, Gwangju 61186, Republic of Korea; Department of Biochemistry, College of Natural Sciences, Chungnam National University, Daejeon 34134, Republic of Korea; Department of Bio and Environmental Technology, College of Science and Convergence Technology, Seoul Women’s University, Seoul 01797, Republic of Korea; School of Biological Science and Technology, Chonnam National University, Gwangju 61186, Republic of Korea; Institute of Systems Biology and Life Science Informatics, Chonnam National University, Gwangju 61186, Republic of Korea

## Abstract

**Motivation:**

Accurate identification of DNA-binding proteins (DBPs) and RNA-binding proteins (RBPs) is critical for elucidating transcriptional and post-transcriptional regulatory mechanisms. However, existing computational approaches often rely on inferred labels or domain-specific annotations, which limit the subsequent generalizability.

**Results:**

This study aimed to introduce transformer-based classifiers for human DBPs and RBPs that rely solely on protein sequence information without engineered features or domain constraints. The models were implemented using ESM-2 with low-rank adaptation (LoRA) fine-tuning and trained on experimentally validated datasets, including chromatin immunoprecipitation sequencing (ChIP-seq) annotations for DBPs and eCLIP annotations for RBPs. Next, to evaluate biological relevance, we computed value-aware attention (VAT) scores aggregated across transformer layers to interpret model focus. In 20-fold cross-validation, the DBP model achieved an area under the receiver operating characteristic curve (AUROC) of 0.84 with a Matthews correlation coefficient (MCC) of 0.40, while the RBP model achieved an AUROC of 0.92 with an MCC of 0.46. Proteins predicted as nucleic acid-binding were enriched for known binding domains, and inspection of attention distributions revealed preferential focus on annotated functional regions rather than non-binding segments. These results demonstrate that attention-based protein language models can accurately identify nucleic acid-binding proteins directly from sequence data. Moreover, these models reveal biologically meaningful sequence determinants of binding, establishing an interpretable and scalable framework for proteome-wide characterization of protein–nucleic acid interactions.

**Availability and implementation:**

Code is available on GitHub (https://github.com/CSB-hub/DRBP).

## 1 Introduction

Nucleic acid-binding proteins regulate transcription, mRNA processing, and translation through sequence-specific or structure-dependent interactions with DNA and RNA (Oliveira *et al.* 2017, Bartas *et al.* 2021). DNA-binding proteins (DBPs), including transcription factors, chromatin remodelers, and DNA repair factors, shape gene expression programs and help maintain genome integrity in response to developmental and environmental cues (Fong *et al.* 2013, Pan *et al.* 2014, Lambert *et al.* 2018). RNA-binding proteins (RBPs) primarily act at the post-transcriptional level, regulating splicing, mRNA stability, subcellular localization, and translation efficiency (Dreyfuss *et al.* 2002, Glisovic *et al.* 2008, Gerstberger *et al.* 2014). Meanwhile, disruption of these DNA- and RNA-binding protein networks has been linked to a broad spectrum of human diseases, including cancer, neurodegenerative disorders, and autoimmune conditions (Conlon and Manley 2017, Kavanagh *et al.* 2022, Li *et al.* 2022). Therefore, accurate identification of nucleic acid-binding proteins is a key step toward a mechanistic understanding of cellular regulation and disease pathology.

Experimental characterization of protein–nucleic acid interactions has advanced through a range of biochemical and genomic techniques (Ray *et al.* 2013, Turner and Díaz-Muñoz 2018, Tak Leung *et al.* 2019). For DBPs, chromatin immunoprecipitation sequencing (ChIP-seq) is widely used to identify genome-wide binding sites in vivo, while protein-binding microarrays (PBMs) enable high-throughput measurements of DNA-binding specificities in vitro (Berger and Bulyk 2006, Jothi *et al.* 2008). For RBPs, crosslinking immunoprecipitation (CLIP) protocols have evolved from the original method to enhanced variants such as PAR-CLIP, iCLIP, and eCLIP, which offer improved resolution and signal-to-noise (Huppertz *et al.* 2014, Spitzer *et al.* 2014, Yang *et al.* 2015, Van Nostrand *et al.* 2016). Despite these advances, proteome coverage from experimental assays remains incomplete, as each experiment typically targets individual proteins and depends on specific antibodies or tagged constructs (Ramanathan *et al.* 2019, Aydin *et al.* 2024). Thus, to complement these efforts, computational methods have been developed to predict DBPs and RBPs directly from protein sequence information. Early approaches relied on handcrafted features such as amino acid composition, physicochemical descriptors, evolutionary profiles, including position-specific scoring matrices, and annotated functional domains (Chen *et al.* 2014, Si *et al.* 2015, Asghari and Abdolmaleki 2019, Pan *et al.* 2020, Li *et al.* 2021, Yan *et al.* 2023). These methods achieved moderate performance but were constrained by the quality of predefined features and by the completeness of available domain annotations. In many cases, these methods also depended on training labels derived from Gene Ontology (GO) annotations or homology-based inference, which can propagate errors from earlier computational predictions and reduce generalizability to proteins outside well-characterized families (Škunca *et al.* 2012, Vu and Jung 2021).

Recent advances in deep learning algorithms have led to the development of protein language models (PLMs) trained on large-scale protein sequence databases using self-supervised objectives such as masked language modeling. Several architectures have been proposed, including the ProtTrans family, which adapts BERT and T5 for protein sequences (Brandes *et al.* 2022, Elnaggar *et al.* 2022), and the ESM series developed by Meta AI (Lin *et al.* 2023, Heinzinger *et al.* 2024, Hayes *et al.* 2025). These models learn contextualized amino acid representations that capture evolutionary and structural patterns without explicit feature engineering (Schmirler *et al.* 2024, Chen *et al.* 2025). Such representations have been applied to a wide range of downstream tasks, including protein structure prediction, variant effect prediction, protein–protein interaction prediction, and subcellular localization (Wang *et al.* 2025, Leclercq and Droit 2026). More recently, PLMs have been used for predicting nucleic acid-binding proteins, and these applications report improved performance compared with traditional feature-based approaches (Zeng *et al.* 2024, Mu *et al.* 2025, Pokharel *et al.* 2025). This shift addresses the principal weakness of feature-based methods, where prediction accuracy is inherently limited by predefined descriptors, by instead learning binding-related signals directly from sequences. However, PLM-based predictors introduce a distinct set of constraints, primarily concerning prediction resolution. On one hand, protein-level classifiers assign a global binding label but lack the granularity to indicate specific sequence regions underlying the prediction. On the other hand, residue-level predictors identify localized candidate binding sites but bypass the prerequisite question of overall nucleic acid-binding capability. As a result, simultaneously achieving accurate protein-level classification and interpretable, region-level attribution remains a challenge for current models. This issue is further compounded when PLM-based models are trained on computationally inferred annotations rather than experimentally verified binding data, thereby inheriting the biases of previous methods.

In addition to improved predictive performance, recent PLMs based on transformer architectures offer new opportunities for model interpretability. Transformer-based models employ attention mechanisms that assign different weights to residues based on the associated contextual relevance within the sequence (Vaswani *et al.* 2017, Chandra *et al.* 2023, Nayar *et al.* 2025). Analysis of these attention patterns can highlight sequence regions that contribute to model predictions, thereby providing residue-level interpretability. In the context of protein function prediction, such attention-based analyses can indicate whether models focus on known functional domains or instead point to previously uncharacterized sequence features associated with the predicted function.

This study aimed to develop transformer-based classification models to predict DBPs and RBPs from human protein sequences. The models were built on ESM-2 with low-rank adaptation (LoRA) fine-tuning and trained on experimentally confirmed DNA- and RNA-binding activities derived from chromatin immunoprecipitation (ChIP) and enhanced crosslinking protocols. In a 20-fold cross-validation, the DBP and RBP models achieved area under the receiver operating characteristic curve (AUROC) values of 0.84 and 0.92, respectively, indicating robust predictive performance on experimentally grounded datasets. To examine whether these predictions are biologically interpretable, we analyzed value-aware attention (VAT) scores and observed that model attention preferentially localizes to annotated nucleic acid-binding domains, including zinc finger and homeodomain regions for DBPs and RNA recognition motifs for RBPs.

## 2 Methods

### 2.1 Data collection and curation

**Figure 1:**
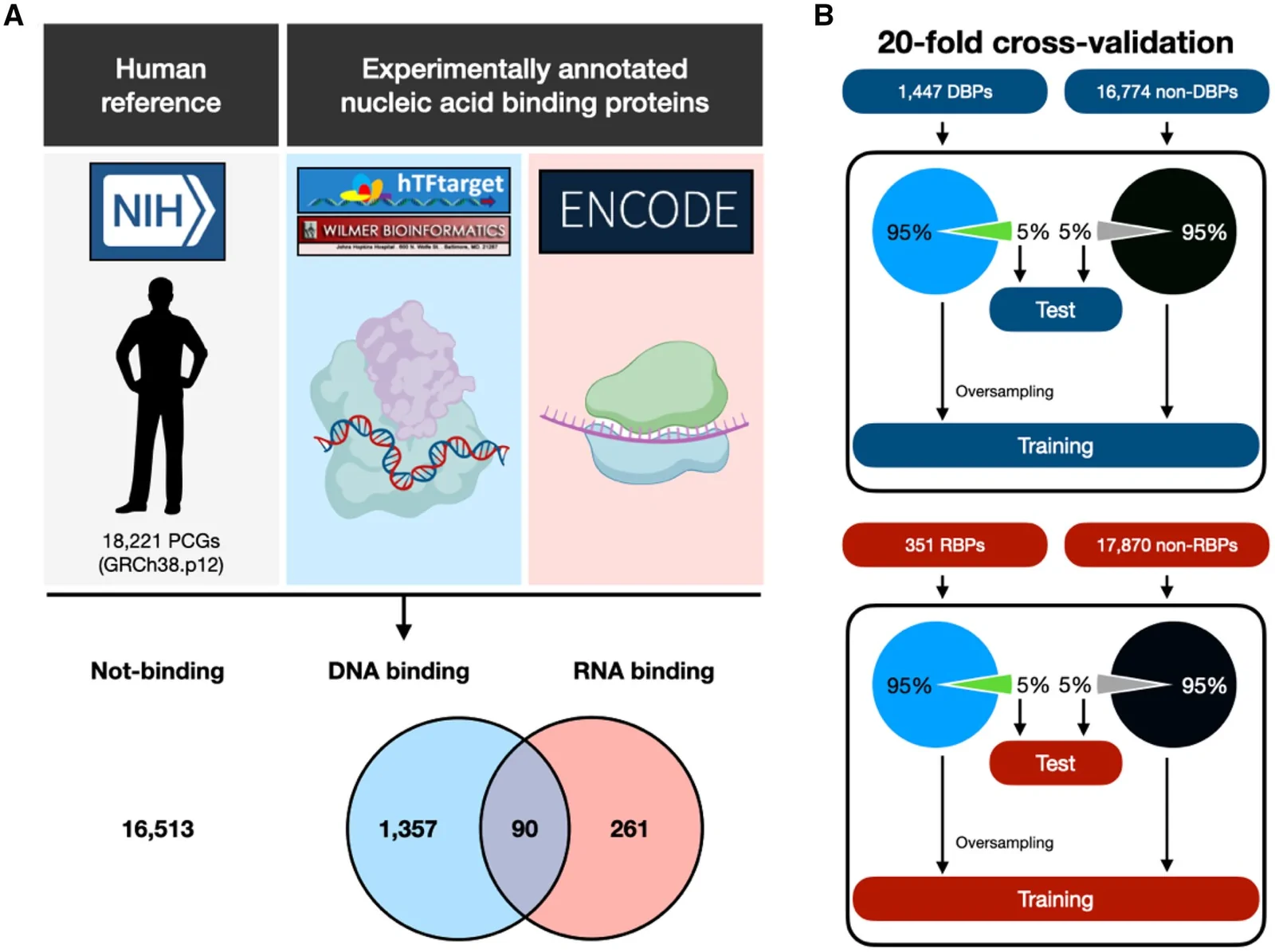
Dataset construction and cross-validation framework. (A) Classification of human protein-coding genes (PCGs; 18 221 genes from GRCh38.p12) based on experimentally validated nucleic acid-binding annotations from hTFtarget, hPDI, and ENCODE. The Venn diagram shows the distribution of DNA-binding proteins (DBPs), RNA-binding proteins (RBPs), dual-binding proteins, and non-binding proteins. (B) Schematic of 20-fold cross-validation and class balancing for DBP and RBP model training.

Human proteins were classified into four distinct categories: DNA-binding, RNA-binding, dual-binding (DNA and RNA), and non-binding. The human reference proteome (GRCh38.p12) was obtained from the Ensembl database (https://www.ensembl.org) and comprises 18 221 protein-coding genes (Fig. 1A). For genes encoding multiple protein isoforms, the longest isoform was selected as the representative sequence. To ensure consistent cross-database identification, only proteins with both Ensembl and SwissProt accession IDs were retained.

DBPs were compiled from two experimentally validated databases: hPDI (Xie *et al.* 2010) and hTFtarget (Zhang *et al.* 2020). The hPDI database contains protein–DNA interaction data derived from microarray assays, including both conventional transcription factors and unconventional DNA-binding proteins. The hTFtarget database provides curated transcription factor-target regulations based on 7190 ChIP-seq samples covering 659 transcription factors across 569 conditions. Integration of these sources yielded 1447 experimentally validated DBPs. RBPs were obtained from the ENCODE project (Van Nostrand *et al.* 2020), which systematically characterized human RBP binding profiles using enhanced crosslinking and immunoprecipitation (eCLIP) and related methods. From this resource, 351 RBPs with both Ensembl and SwissProt accession IDs were identified.

The prediction framework was designed as two independent binary classification tasks. For each task, proteins with experimentally validated binding activity were designated as positive samples, while all remaining proteins without experimental evidence were conservatively assigned as negatives. For DBP prediction, the 1447 validated DBPs served as positives and the remaining 16 774 proteins as negatives. For RBP prediction, the 351 confirmed RBPs constituted the positive class, and the remaining 17 870 proteins were assigned as the negative class. The smaller size of the RBP positive set reflects its reliance on a single experimental repository (ENCODE eCLIP) and our strict exclusion of computationally inferred annotations to ensure data quality.

### 2.2 Input sequence preparation for model training

The input sequence length was set to 1024 amino acids based on constraints imposed by the ESM-2 model and sequence coverage analysis (Fig. 1, available as supplementary data at *Bioinformatics Advances* online). This threshold captured approximately 91% of DNA-binding sequences and 88% of RNA-binding sequences, with comparable coverage for non-binding sequences (87%–88%). Sequences exceeding this length were truncated from the C-terminus, while shorter sequences were zero-padded at the C-terminus.

### 2.3 Model architecture

Protein sequences were encoded using the pretrained protein language model ESM-2 (esm2_t33_650M_UR50D), an encoder-only transformer comprising 33 self-attention layers with a hidden dimension of 1280 and approximately 650 million parameters (Lin *et al.* 2022). Input sequences were tokenized using the original ESM-2 tokenizer. A classification head, consisting of a task-specific feedforward layer with sigmoid activation, was added on top of the transformer encoder to produce binary predictions.

To enable parameter-efficient training, LoRA (Hu *et al.* 2022) was applied with the following configuration: rank (*r*) = 8, scaling factor (lora_alpha) = 32, dropout rate = 0.1, and target modules = query, key, and value projection layers. The classifier layer was excluded from LoRA and trained with full parameter updates. These rank and scaling parameters were selected based on established configurations in previous protein language model studies (Sledzieski *et al.* 2024, Clancy *et al.* 2025) rather than through task-specific hyperparameter optimization.

### 2.4 Training and validation strategies

Model training was implemented in PyTorch using the Hugging Face Transformers library with the PEFT module. Binary cross-entropy with logits was used as the loss function. The AdamW optimizer was used with a learning rate of 1 × 10^−5^ and a batch size of 16. Training was conducted for five epochs with early stopping triggered when validation loss did not improve for three consecutive iterations. All experiments were performed on an NVIDIA GeForce RTX4090 GPU.

Model performance was evaluated using 20-fold cross-validation. To address class imbalance, positive samples were up-sampled by each sample 12-fold for DBP prediction and 50-fold for RBP prediction to approximate the size of the negative set. In each iteration, one partition (5% of the data) was held out for evaluation while the remaining 95% was used for training. This allocation allowed the RBP task to retain approximately 333 out of 351 positive proteins per iteration, ensuring a sufficient training size while providing out-of-fold predictions for all samples. This configuration was selected to maximize training data for minority class without introducing the evaluation variance and computational cost associated with a larger number of data splits.

### 2.5 Model performance assessment

Model performance was evaluated using multiple metrics to ensure a comprehensive assessment under conditions of class imbalance. The primary evaluation metric was the MCC, calculated as:


MCC=((TP×TN–FP×FN)/√((TP+FP)(TP+FN)(TN+FP)(TN+FN)))

where TP, TN, FP, and FN represent true positives, true negatives, false positives, and false negatives, respectively. Additional metrics included accuracy (TP + TN)/(TP + TN + FP + FN), balanced accuracy ([TP/(TP + FN) + TN/(TN + FP)]/2), precision [TP/(TP + FP)], recall [TP/(TP + FN)], specificity [TN/(TN + FP)], and F1-score [2 × precision × recall/(precision + recall)]. Receiver operating characteristic (ROC) curves were generated, and the area under the curve (AUC) was calculated to assess overall discriminative performance. All metrics were averaged across the 20 folds to obtain robust estimates. The optimal classification threshold for each model was determined by selecting the probability cutoff that maximized the MCC across validation folds. This approach was chosen as MCC provides a balanced measure even when class distributions are highly skewed.

To ensure a direct benchmark, ESM-DBP (Zeng *et al.* 2024) and TransBind (Tahmid *et al.* 2025) were selected as the protein-level baselines for the DBP task, while DRBP-EDP (Mu *et al.* 2025) and HydRA (Jin *et al.* 2023) were selected for the RBP task. These alternative methods were evaluated under the same computational framework using the complete dataset constructed in this study, thereby maintaining identical protein sequences, ground-truth labels, and metric definitions.

### 2.6 Protein domain annotation and enrichment analysis

Protein domains were annotated for 18 221 human protein sequences using InterProScan (version 5.73-104.0) (Jones *et al.* 2014). A total of 387 985 annotation records were generated, and only entries associated with IPR signatures were retained for downstream analyses.

InterProScan integrates predictions from multiple member databases, such as Pfam, SMART, ProSiteProfiles, TIGRFAMs, and SUPERFAMILY, which often result in overlapping or partially redundant annotations for the same protein region. A consensus interval was derived for each IPR to resolve inconsistencies in domain boundaries. The consensus interval for each IPR was determined using two criteria: (i) when multiple databases assigned differing boundaries to the same IPR, the interval supported by the largest number of annotations was selected; (ii) overlapping intervals belonging to the same IPR were merged when at least one residue was shared.

To characterize domain composition, the distribution of IPR domain counts and the proportion of each IPR were analyzed separately for predicted positive and predicted negative groups. The VAT scores for the IPR domains were compared between predicted positive and predicted negative groups using the Mann–Whitney *U* test.

### 2.7 Attention-based interpretation analysis

To interpret model predictions, the VAT scores were used to evaluate residue-level importance, as previously described (Guo *et al.* 2024). VAT was calculated by multiplying the attention weight by the L2 norm of the corresponding value vector. VAT scores were extracted from all transformer layers and averaged across the ESM-2 model. For models with multiple attention heads, attention scores were averaged across all heads. Scores were computed for each cross-validation fold and averaged across folds.

A pronounced accumulation of VAT scores near the [CLS] token was identified as position-dependent bias. To mitigate this effect, a cubic smoothing spline was applied to the model to remove the position-dependent baseline bias along the protein sequence. A UnivariateSpline was fitted to model the position-dependent background, with the smoothing factor optimized to capture the global baseline pattern without overfitting to local fluctuations. Subsequently, sequence-level *z*-score normalization was applied by subtracting the mean and dividing by the standard deviation of VAT scores within each protein to quantify the relative importance of residues. Whole-sequence *z*-score normalization was also performed to enable comparison of VAT score distributions across different proteins.

For baseline control, VAT scores were also extracted from the untuned ESM-2 architecture (esm2_t33_650M_UR50D) without LoRA fine-tuning. The baseline profiles were generated using the identical calculation and normalization parameters applied to the fine-tuned models, allowing a direct comparison of attention distributions across the same representative proteins.

## 3 Results

### 3.1 Dataset construction and model training configuration

We constructed a dataset of nucleic acid-binding proteins from 18 221 human protein-coding genes (GRCh38.p12) by integrating experimentally validated annotations from hPDI, hTFtarget, and ENCODE databases (Fig. 1A). Importantly, all positive annotations were derived exclusively from experimental evidence, including ChIP-seq data for DBPs and CLIP-seq data for RBPs, rather than from computational predictions or sequence-homology-based inference. This approach identified 1447 DBPs and 351 RBPs, with 90 proteins exhibiting dual-binding capacity for both DNA and RNA. For binary classification, proteins without experimental evidence for DNA binding (*n* = 16 774) and RNA binding (*n* = 17 870) were designated as negative classes for the DBP and RBP models, respectively.

For model input, protein sequences were truncated to a maximum length of 1024 residues to balance sequence coverage with computational efficiency. This threshold yielded retention efficiencies of 91% for DBPs, 88% for RBPs, and 87%–88% for non-binding proteins (Fig. 1, available as supplementary data at *Bioinformatics Advances* online). We employed a 20-fold cross-validation framework in which the dataset was split into 95% training and 5% test sets per fold (Fig. 1B). Since both tasks exhibited substantial class imbalance, positive samples were oversampled within the training sets by factors of 12 for DBPs and 50 for RBPs. The test sets retained the original class distributions, ensuring an unbiased evaluation of model performance.

### 3.2 The ESM-2-based prediction model achieves high performance and outperforms existing predictors for both DBPs and RBPs

**Figure 2:**
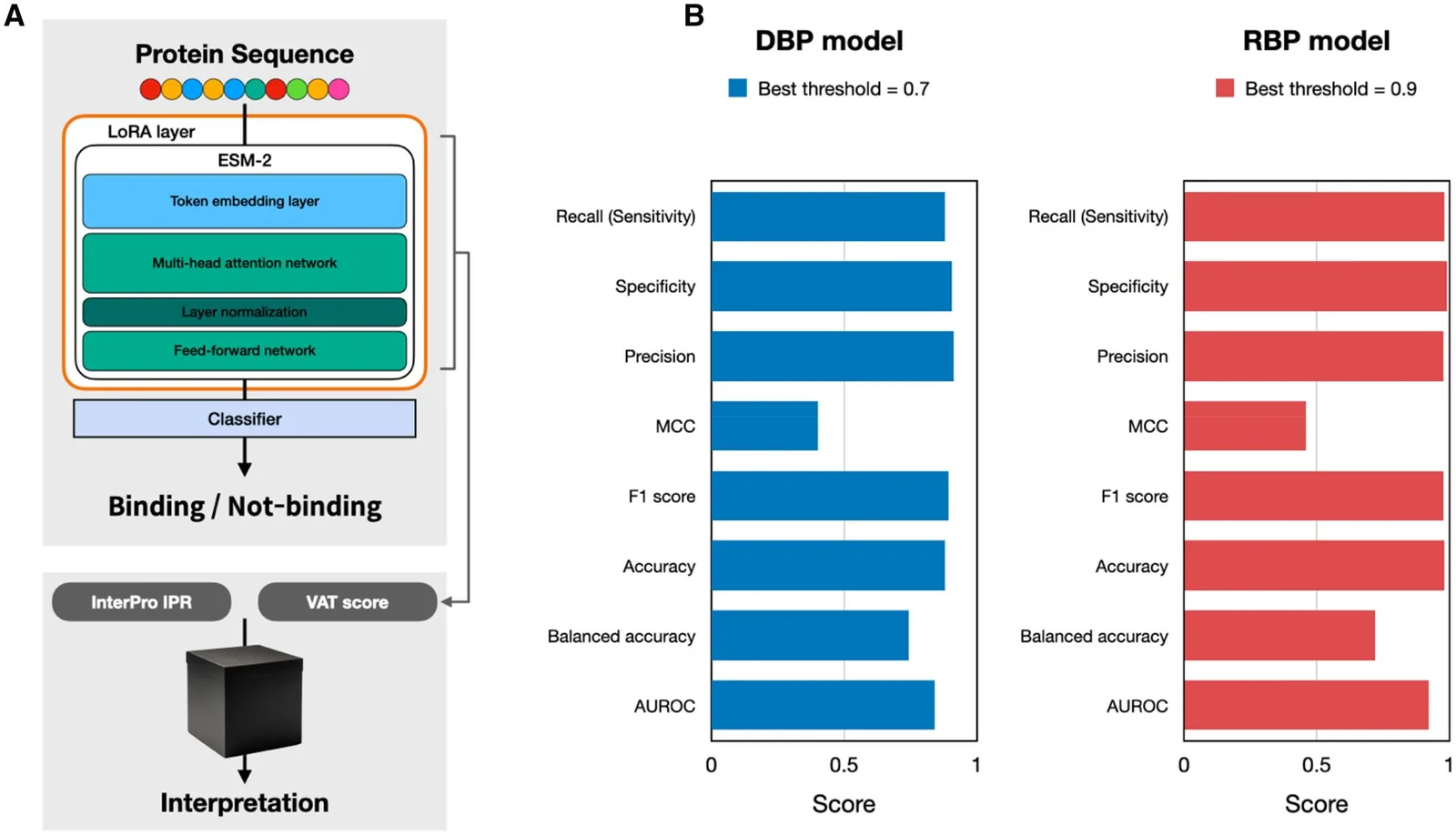
Model architecture and classification performance. (A) Schematic of the ESM-2 protein language model with low-rank adaptation (LoRA) fine-tuning for nucleic acid-binding protein classification. Value-aware attention (VAT) scores enable interpretability analysis. (B) Performance metrics for the DBP model (left) and RBP model (right). Optimal thresholds of 0.7 for DBP and 0.9 for RBP were determined based on the maximum Matthews correlation coefficient (MCC). The area under the receiver operating characteristic (AUROC) curve is presented.

We developed transformer-based classification models using ESM-2 with LoRA fine-tuning for DBP and RBP predictions (Fig. 2A). To determine optimal classification thresholds, we systematically evaluated model performance across probability cutoffs, ranging from 0.0 to 1.0 (Fig. 2A, available as supplementary data at *Bioinformatics Advances* online). Multiple metrics were assessed, including accuracy, precision, recall, F1-score, and Matthews correlation coefficient (MCC), with the optimal threshold selected by maximizing the MCC, which provides a balanced measure of performance under imbalanced class conditions. The DBP model achieved the highest MCC at a threshold of 0.7, whereas the RBP model optimized at a higher threshold of 0.9. This variation directly reflects the differing degrees of class imbalance between tasks; because the RBP dataset contains a substantially smaller proportion of positive samples, a more stringent probability cutoff was required to suppress false-positive predictions.

At these optimized thresholds, both models demonstrated strong predictive performance in a 20-fold cross-validation (Fig. 2B). The DBP model achieved an accuracy of 0.88, an MCC of 0.40, and an AUROC of 0.84. The RBP model achieved an accuracy of 0.98, MCC of 0.46, and AUROC of 0.92. Meanwhile, applying the trained models to the full dataset of 18 221 human proteins revealed distinct prediction distributions (Fig. 2B, available as supplementary data at *Bioinformatics Advances* online). The DBP model identified 2412 proteins with predicted binding probabilities above 0.7, and the RBP model predicted 529 proteins with predicted binding probabilities above 0.9 as potential binding proteins.

We next benchmarked our framework against existing protein-level predictors on the same dataset (Table 1). The DBP model outperformed the baselines across major metrics, achieving an MCC of 0.40 and AUROC of 0.84, which exceeded the values obtained by ESM-DBP (0.343 and 0.812, respectively) and TransBind. Similarly, the RBP model achieved a higher MCC than DRBP-EDP (0.361) and HydRA (0.251), although DRBP-EDP showed a marginally higher AUROC (0.933 vs. 0.920) and both baselines showed marginally higher precision.

**Table 1:** Benchmark comparison of DBP and RBP prediction performance.

Task	Model	Recall	Specificity	Precision	F1-score	MCC	AUROC
DBP	This study	**0.881**	**0.906**	**0.913**	**0.894**	**0.400**	**0.840**
	ESM-DBP	0.831	0.847	0.911	0.861	0.343	0.812
	TransBind	0.374	0.386	0.789	0.494	−0.207	0.262
RBP	This study	**0.980**	**0.990**	0.979	**0.979**	**0.460**	0.920
	DRBP-EDP	0.931	0.934	0.980	0.951	0.361	**0.933**
	HydRA	0.791	0.788	**0.982**	0.867	0.251	0.883

Bold values indicate the best-performing result for each metric among the compared models within each task.

### 3.3 Domain enrichment analysis reveals canonical binding domains in predicted proteins

**Figure 3:**
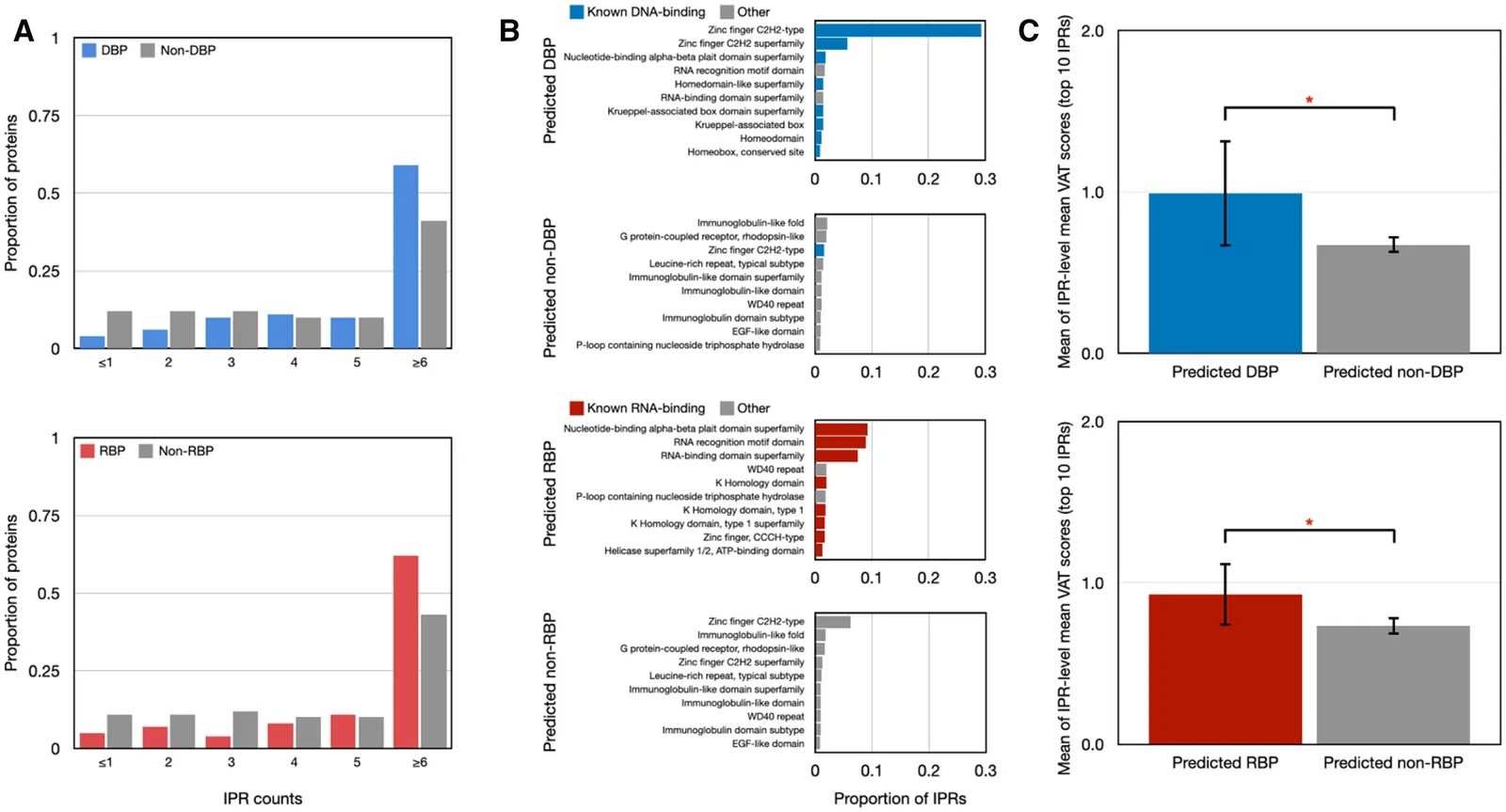
Domain enrichment analysis and attention-based interpretation. (A) Distribution of InterPro (IPR) domain counts in predicted DBPs versus non-DBPs (top) and RBPs versus non-RBPs (bottom). The x-axis represents the number of annotated IPR domains per protein. (B) Top 10 enriched IPR domains in each predicted group. Known DNA-binding or RNA-binding domains are highlighted. (C) Comparison of mean of IPR-level mean VAT scores across the top 10 IPR domains between positive and negative prediction groups. Error bars represent standard error. Statistical significance was determined by the Mann–Whitney *U* test (*P < .05).

To assess whether the predicted binding proteins exhibited biologically interpretable features, we examined InterPro (IPR) domain annotations (Fig. 3, available as supplementary data at *Bioinformatics Advances* online). Comparison between binding and non-binding predicted groups showed no consistent pattern for proteins with fewer than six IPR domains; however, proteins containing six or more domains were notably more prevalent in the binding groups for both models (Fig. 3A). These findings suggest that IPR domain profiles provide useful insights for the biological interpretation of nucleic acid-binding protein predictions.

Next, we examined which specific IPR domains were enriched in each predicted group (Fig. 3B). In the DBP-positive group, zinc finger C2H2-type domains, homeobox-related domains, and other DNA-binding-associated motifs were among the most enriched IPRs. The RBP-positive group showed enrichment for RNA-binding-related domains, including RNA recognition motifs (RRMs), KH domains, and RNA-binding domain superfamilies. Conversely, proteins predicted as non-binding were enriched for broadly distributed or signaling-related domains, such as immunoglobulin-like folds and G protein-coupled receptor (GPCR) domains.

These observations led us to investigate whether the attention patterns for the model reflected enrichment for binding-associated domains. We quantified VAT scores within regions corresponding to the top 10 IPR domains and found that the mean VAT scores were significantly higher in the positive predicted groups than in the negative groups for both the DBP and RBP models (Fig. 3C; *P* = .011 and *P* = .017, respectively). These results demonstrate that the model assigns greater attention to regions of the domain associated with nucleic acid-binding functions, consistent with the domain enrichment patterns observed in the predicted binding groups.

### 3.4 Attention-based analysis identifies functional binding regions

**Figure 4:**
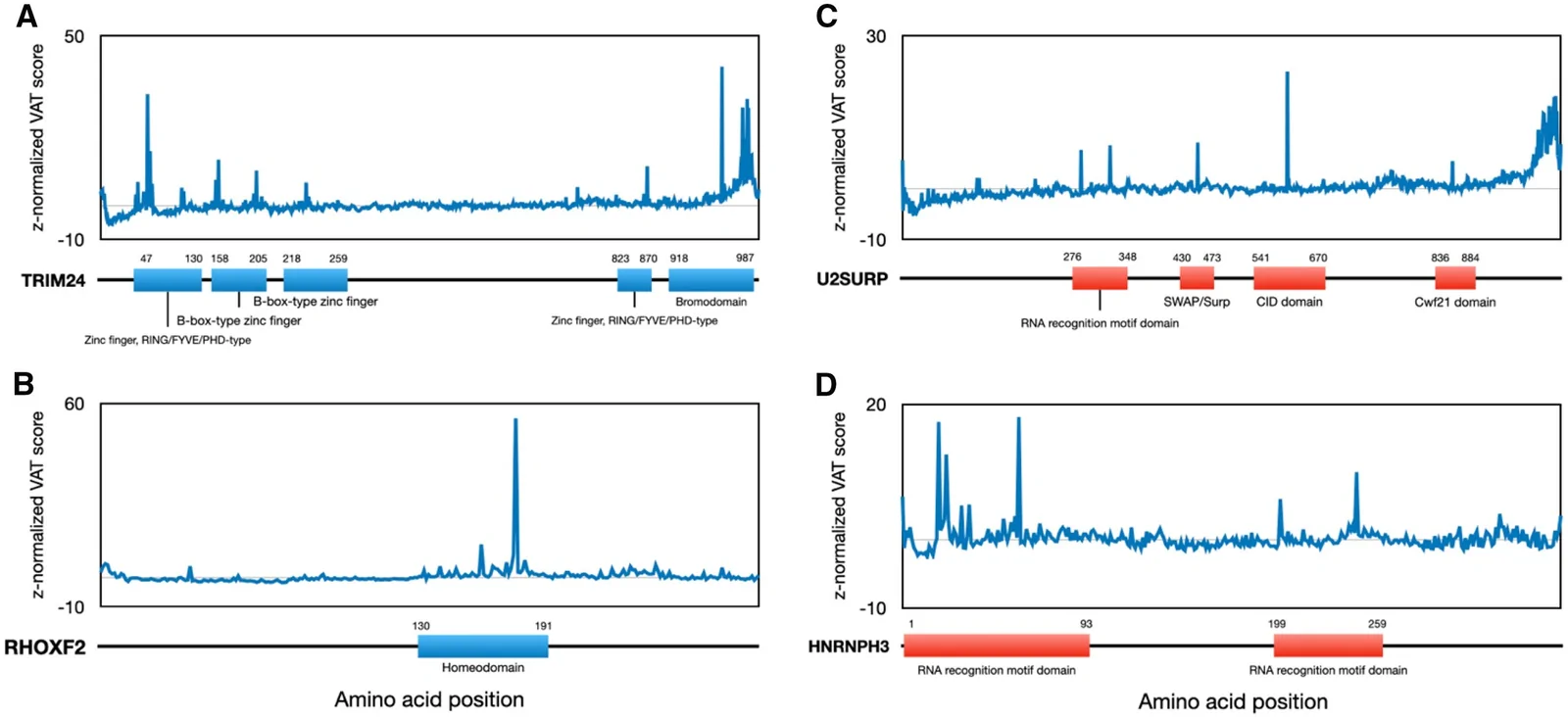
Representative value-aware attention score profiles from the fine-tuned models for DNA- and RNA-binding proteins. (A, B) VAT score profiles for DBPs: TRIM24 (A) and RHOXF2 (B). (C, D) VAT score profiles for RBPs: U2SURP (C) and HNRNPH3 (D). The *x*-axis represents amino acid position, and the *y*-axis represents z-normalized VAT scores. Annotated protein domains are shown below each profile. Annotated DBP and RBP domains are shown below each profile.

To visualize how the attention of the model is distributed across functional domains at the individual protein level, we present the VAT score profiles for proteins predicted to be nucleic acid-binding (Fig. 4). For the DBP predictions, we selected proteins with distinct domain architectures. TRIM24 contains multiple zinc finger and bromodomain regions, and RHOXF2 harbors a single homeodomain. Despite these structural differences, both proteins exhibited VAT score peaks concentrated over annotated DNA-binding domains, with lower scores in regions lacking binding annotations (Fig. 4A and B).

A consistent pattern was observed in the RBP predictions. U2SURP contains multiple RNA-binding-associated domains, including RRM, SWAP/Surp, CID, and Cwf21 domains, and showed VAT score peaks corresponding to each domain region (Fig. 4C). HNRNPH3 contains two RRM domains, while the VAT peaks coincided specifically with these annotated regions (Fig. 4D). These examples confirm that the attention for the model corresponds to annotated nucleic acid-binding domains at the single-protein level, as demonstrated in the domain-level analysis.

We next evaluated whether these localized attention patterns depended on fine-tuning by comparing the VAT profiles with the untuned ESM-2 based model (Fig. 4 and Fig. 4, available as supplementary data at *Bioinformatics Advances* online). The untuned model displayed a similar preference for annotated DNA- or RNA-binding domains prior to optimization. The peak coordinates within these functional regions closely matched those obtained from the fine-tuned models across the representative proteins, confirming that these domain-level features are intrinsic to the pretrained sequence representations.

## 4 Discussion and conclusion

The models developed in this study classify nucleic acid-binding proteins from sequence alone and localize attention to regions that underpin these classifications. Meanwhile, to understand how the model encodes these functional signals, we performed systematic domain enrichment and attention-based analyses and found that attention is preferentially concentrated within annotated DNA- and RNA-binding domains across diverse protein architectures. These properties indicate that the model can extract domain-level signals directly from the sequence, without relying on explicit prior knowledge of binding regions. In this respect, our approach departs from traditional methods for predicting DBPs and RBPs, which typically involve explicit domain annotations, physicochemical properties, or manually engineered features to characterize nucleic acid-binding proteins (Chen *et al.* 2014, Si *et al.* 2015, Pan *et al.* 2020, Li *et al.* 2021). Instead, our model uses only protein sequence information as input, without predefined structural or functional descriptors, yet still recognizes and exploits domain-level signals relevant to nucleic acid binding, as evidenced by the preferential attribution of attention to known functional regions. This observation suggests that the pretrained representations of ESM-2 inherently encode information about functionally important protein regions, consistent with previous studies demonstrating that protein language models capture evolutionary and structural patterns from sequence data alone (Lin *et al.* 2023).

Although several PLM-based methods have recently been developed to predict nucleic acid-binding functions, existing approaches vary considerably in prediction granularity, data provenance, and interpretability. For instance, protein-level classifiers such as ESM-DBP (Zeng *et al.* 2024) rely on potentially biased, computationally inferred GO annotations. In contrast, structure-based residue-level predictors, including EquiPNAS (Roche *et al.* 2024) and CLAPE-DB (Liu and Tian 2025), identify binding sites using the BioLiP database but lack mechanisms to map these sites onto functional domains. Even sequence-based hybrid models like TransBind (Tahmid *et al.* 2025) incorporate PDB-derived labels to address both resolutions, yet a unified implementation remains absent. Across these methods, residue-level predictors and protein-level classifiers each address only one side of the problem, so accurate protein-level classification and interpretable, region-level attribution are seldom achieved together. Our framework addresses this gap by jointly performing the protein-level classification of DNA- and RNA-binding activity and region-level interpretation via VAT scores, which highlight the sequence segments most influential for individual predictions. This integrated design enables accurate detection of nucleic acid-binding proteins and provides a basis for formulating hypotheses regarding the functional domains that mediate binding. In addition, we adopted LoRA fine-tuning, a parameter-efficient adaptation strategy that regularizes training and mitigates overfitting when large PLMs are trained on limited data, thereby substantially reducing the number of parameters that must be updated.

Our methodology is further distinguished by the exclusive use of high-quality, experimentally verified datasets from diverse sources, including microarray assays, ChIP-seq, and eCLIP-based functional studies. Many existing DBP and RBP prediction methods, including recent transformer-based approaches, are trained on datasets constructed from GO annotations, structure-based repositories such as BioLiP, or other computationally inferred labels. These sources can introduce noise through in silico annotation and lead to circular validation, in which models learn to reproduce biases from earlier prediction tools rather than true biological patterns (Schnoes *et al.* 2013). In contrast, this model relies exclusively on experimentally supported annotations: hPDI for protein–DNA interactions, verified by microarray assays; hTFtarget for transcription factor–target relationships, confirmed by ChIP-seq across 7190 samples, and ENCODE for RBPs characterized using stringent eCLIP protocols. This design encourages the model to learn genuine sequence–function relationships and reduces the risk of false positives that are common in models trained on purely computational predictions. Consequently, the performance metrics reported in this study are more likely to capture true generalization capability rather than artifacts arising from contaminated training labels.

Beyond predictive performance, interpretability analysis indicates that functional domains associated with nucleic acid binding heavily influence the classification mechanisms. Attention-based scores were preferentially elevated within annotated domain regions along the protein sequence, demonstrating that the learned representations capture functionally relevant, domain-level features. The tight correspondence between VAT score peaks and annotated domains, valid in both enrichment statistics and individual test cases (TRIM24, RHOXF2, U2SURP, and HNRNPH3), suggests that the model relies on structural signatures encoded natively by ESM-2 from sequence alone. A direct comparison with the untuned ESM-2 model reinforces this conclusion. The base model displayed the same preferential attention to annotated binding domains as its fine-tuned counterparts, with nearly identical peak alignments. These results confirm that the domain-level signals captured by the VAT scores are inherent to the pretrained representations rather than being induced de novo during downstream training. LoRA fine-tuning simply calibrates these preexisting sequence representations to optimize protein-level classification without disrupting their structural interpretability. These findings have implications for the discovery of novel nucleic acid-binding proteins. Meanwhile, proteins that receive high prediction scores but lack annotations in curated databases may be promising candidates for experimental validation, particularly when the attention profiles of these candidates are concentrated in specific sequence regions. In this setting, the framework can serve as a hypothesis-generating tool for identifying previously uncharacterized binding proteins or unconventional binding mechanisms. Meanwhile, attention weights should be interpreted with caution, as these weights reflect correlation rather than causation (Serrano and Smith 2019). High attention scores indicate that certain residues are associated with the predictions of the model, but do not, by themselves, show that these residues are necessary or sufficient for binding activity. Nevertheless, the consistent correspondence between attention patterns and independently annotated functional domains supports the biological relevance of the learned representations.

Future work could extend and validate these findings in several directions. Experimental approaches, such as conducting site-directed mutagenesis at the residues with high attention scores, would provide direct evidence for the functional importance of regions highlighted by the model. In addition, proteins predicted to be nucleic acid-binding but absent from current reference databases constitute candidates for biochemical validation using techniques such as electrophoretic mobility shift assays or crosslinking immunoprecipitation. From a computational standpoint, the present binary classification framework could be extended to a multi-task setting that jointly predicts DNA-binding, RNA-binding, and dual-binding capabilities; for example, incorporating protein structural information from AlphaFold2-predicted models (Bryant *et al.* 2022, Wei *et al.* 2022, Yuan *et al.* 2022, Bertoline *et al.* 2023) may further enhance predictive performance and enable residue-level binding-site predictions rather than protein-level classifications. The same framework could also be used to assess how disease-associated variants influence nucleic acid-binding functions, thereby contributing to the interpretation of variants of uncertain significance in clinical genomics.

Several limitations of this study should be acknowledged. First, the experimentally validated positive datasets, particularly for RBPs (*n* = 351), are relatively small compared with the negative class, which may limit the ability of the model to capture the full diversity of binding mechanisms. Second, the focus on experimentally verified data may introduce a bias toward well-studied proteins, as proteins from less-characterized families or with context-dependent binding activities are underrepresented in current experimental resources. Third, the negative class in this study comprises proteins for which there is no experimental evidence of nucleic acid binding, rather than proteins conclusively shown to lack binding activity; some of these proteins may possess binding functions that have not yet been characterized. To mitigate this uncertainty, we employed 20-fold cross-validation to distribute potential hidden positives across folds and reduce the influence of these data on performance estimates. Consistent with this concern, a subset of proteins classified as false positives by the model was independently annotated as DNA- or RNA-binding in UniProt. This suggests that some of these predictions may actually be genuine nucleic acid-binding proteins that are not included in the curated reference datasets used for training and evaluation. Although cross-validation is robust to random label noise, this intrinsic property of the dataset may still introduce a modest bias in overall performance estimates. Finally, the sequence-length constraint of 1024 residues resulted in the truncation of 9%–13% of proteins, and the model was trained exclusively on human proteins, indicating that additional validation will be necessary before applying the approach to other species.

In conclusion, this study addresses the challenge of developing predictive models that are accurate, interpretable, and supported by reliable experimental evidence. The correspondence between attention patterns and established functional domains provides a basis for relating machine learning predictions to underlying biological mechanisms. This strategy is broadly applicable to other protein function prediction tasks in which interpretability is important for experimental validation and biological discovery. As experimental resources continue to grow and deep learning architectures advance, sequence-based models trained on high-confidence annotations are likely to become increasingly important for characterizing protein–nucleic acid interactions at the proteome scale.

## Supplementary Material

vbag235_Supplementary_Data

## Data Availability

The authors confirm that the data supporting the findings of this study are available in the article and the associated supplementary materials. All scripts for data preprocessing, model training, and evaluation are available at https://github.com/CSB-hub/DRBP. Trained model weights are provided in the repository. The repository includes detailed instructions for reproducing the analyses reported in this study.
