# Supplementary material for: Interpretable prediction of nucleic acid-binding proteins using a protein language model
Source: Bioinform Adv. 2026 Aug 17;6(1):vbag235. doi: 10.1093/bioadv/vbag235 (PMC13548431; doi:10.1093/bioadv/vbag235)
Supplement: Supplementary file 1 [file vbag235_supplementary_data.docx]

Figure S1. Sequence length distribution and coverage analysis. Distribution of protein sequence lengths for binding-labeled proteins (A) and non-binding-labeled proteins (B). The dashed vertical line indicates the input size cutoff of 1,024 amino acids. With this cutoff, 91% of DNA-binding proteins (DBPs) and 88% of RNA-binding proteins (RBPs) are fully covered in the binding-labeled groups, and 87% of non-DBPs and 88% of non-RBPs are fully covered in the non-binding-labeled groups.

Alt text: Overlaid histograms of protein sequence lengths for DBPs and RBPs in panel A and non-DBPs and non-RBPs in panel B. In both panels, most proteins are shorter than the dashed 1,024-amino-acid cutoff. The cutoff fully covers 91% of DBPs, 88% of RBPs, 87% of non-DBPs, and 88% of non-RBPs.

Figure S2. Threshold optimization and prediction score distribution. (A) Performance metrics across different classification thresholds for the DBP model (top) and RBP model (bottom). The dashed vertical lines indicate optimal thresholds of 0.7 for DBPs and 0.9 for RBPs, determined by the maximum Matthews correlation coefficient (MCC). (B) Distributions of predicted binding probabilities for all proteins. The dashed vertical lines indicate the optimal thresholds, with positive predicted groups shown to the right.

Alt text: Threshold-dependent performance and prediction probability distributions for the DBP and RBP models. Panel A shows accuracy, balanced accuracy, precision, recall, specificity, F1 score, and MCC across classification thresholds. MCC reaches its maximum at 0.7 for the DBP model and 0.9 for the RBP model. Panel B shows that most proteins have low predicted binding probabilities, with smaller groups exceeding the selected positive thresholds.

Figure S3. Workflow for domain enrichment and attention-based interpretability analysis. Model-predicted positive and negative groups are subjected to domain annotation using InterProScan. Group-enriched InterPro (IPR) domains are identified, and the associated annotated positions are used to analyze the value-aware attention (VAT) score.

Alt text: Left-to-right workflow for domain enrichment and attention-based interpretation. Model predictions are separated into positive and negative groups, protein domains are annotated using InterProScan, group-enriched InterPro domains and their sequence positions are identified, and value-aware attention scores are analyzed within the corresponding domain regions.

Figure S4. Representative value-aware attention score profiles from the untuned ESM-2 models. VAT scores were computed from the untuned ESM-2 model (esm2_t33_650M_UR50D) without LoRA fine-tuning. Panels (A–D) and all annotations are as in Figure 4.

Alt text: Four line plots showing z-normalized value-aware attention scores from the untuned ESM-2 model along the sequences of TRIM24, RHOXF2, U2SURP, and HNRNPH3. Annotated DNA- and RNA-binding domains are displayed beneath the profiles. Attention peaks occur at or near several of the same zinc-finger, bromodomain, homeodomain, and RNA recognition motif regions highlighted by the fine-tuned models in Figure 4.
